# Influence of Heat Treatment Parameters of Austempered Ductile Iron on the Microstructure, Corrosion and Tribological Properties

**DOI:** 10.3390/ma16114107

**Published:** 2023-05-31

**Authors:** Halina Krawiec, Janusz Lelito, Marek Mróz, Magdalena Radoń

**Affiliations:** 1 Faculty of Foundry Engineering, AGH University of Science and Technology, Al. A. Mickiewicza 30, 30-059 Kraków, Poland; 2 Department of Foundry and Welding, Rzeszow University of Technology, Powstańców Warszawy 12, 35-959 Rzeszow, Poland; mfmroz@prz.edu.pl (M.M.); m.radon@prz.edu.pl (M.R.)

**Keywords:** austempered ductile iron, microstructure, scratch test, corrosion

## Abstract

The influence of heat treatment parameters such as the annealing time and austempering temperature on the microstructure, tribological properties and corrosion resistance of ductile iron have been investigated. It has been revealed that the scratch depth of cast iron samples increases with the extension of the isothermal annealing time (from 30 to 120 min) and the austempering temperature (from 280 °C to 430 °C), while the hardness value decreases. A low value of the scratch depth and a high hardness at low values of the austempering temperature and short isothermal annealing time is related to the presence of martensite. Moreover, the presence of a martensite phase has a beneficial influence on the corrosion resistance of austempered ductile iron.

## 1. Introduction

Currently, machine and car elements the world over are produced in great numbers and are operated under heavy loads, often, in corrosive environments. The aim is to constantly increase their durability and energy efficiency. The elements that make up these machines and cars, that are currently mainly comprised of alloy steels, are subjected to high loads and are exposed to high abrasive wear and seizure, as well as surface fatigue wear, which is the cause of many machine failures. For these reasons, some steel castings are replaced by austempered ductile iron (ADI) castings.

Austempered ductile iron is one of the latest developments in iron–carbon foundry alloys. The possibility of obtaining a several times greater strength and several times greater lifespan of cast iron by only changing the form of flake graphite to nodular graphite was the reason for avoiding a heat treatment for years, mainly for economic reasons. Now, however, due to the search for methods of further increasing the strength and plasticity of cast iron castings while also giving them special properties that are impossible to achieve with the use of other cast iron alloys, heat treatment is becoming an important development factor in the production of castings from this alloy. Only with the spherical form of graphite can the properties of the matrix be used to the maximum extent [1].

Hardening with an isothermal transformation of ductile iron is completed via heating the castings to the appropriate austenitizing temperature, i.e., in the range from 850–950 °C and holding at this temperature for the appropriate time needed to transform the entire structure into austenite [1,2,3,4], then cooling in an environment that maintains a constant temperature in the range of 250–400 °C [2,3,4] and then holding at this temperature for the desired transformation time [1]. The main goal of isothermal quenching in nodular cast iron is to achieve a characteristic matrix structure consisting of a mixture of ferrite and high-carbon austenite. The share of the latter may be in the range of 5–40% [5,6,7,8,9,10,11,12] and, thanks to such a large amount of this phase in the matrix, ADI cast iron is hardened by impact, crushing or machining, similar to Hadfield’s cast steel [10,11,13]. Until recently, such a structure was often referred to as bainitic [2,3,4,5,14] but is now defined as ausferrite [1,6,7,13].

Ductile iron hardening with isothermal transformation (ADI), also known as ausferritic ductile iron, has been recognized as an important engineering material due to its strength and plastic properties [2,3,4,5,6,7,8,9,10,13,15,16,17,18,19,20,21,22,23,24,25,26,27,28,29,30,31,32,33,34,35], good abrasion resistance and good fatigue strength [20,36], and, due to the presence of spherical graphite in the structure, its good ability to dampen vibrations and satisfactory machinability [13]. Moreover, castings made of ADI cast iron are characterized by a lower density of about 10% compared to steel or cast steel [13]. Thanks to these advantages, ADI cast iron has become an attractive material for, among others, the automotive, agriculture and energy industries [1,16,26], and is used mainly for crankshafts, replacing steel forgings [10,13].

The corrosion behavior of ADI cast iron in electrolyte solutions was presented in several publications [37,38,39,40,41]. These studies described the influence of temperature and isothermal resistance time, microstructure, and physicochemical parameters (e.g., ion nature, temperature, and pH) on the corrosion behavior of ADI cast iron. In the publication [42], the authors presented preliminary studies on the corrosive behavior of ADI cast iron under salt spray conditions. More information on this subject can be found in the publication authored by H. Krawiec et al. [43], where the corrosive behavior of cast iron (ADI) under the influence of cyclic exposure to salt spray was investigated using a special three-electrode cell placed in a salt spray chamber. Three stages were identified, namely, ferrite pitting followed by internal oxidation of the ausferritic matrix, and then a mixed oxidation of the matrix associated with the formation of a rust layer at the sample surface. The corrosion rate evolution calculated from in situ electrochemical measurements was correlated with quantified surface changes. Corrosion products have also been identified.

The main aim of this work is to investigate the effect of both the austempered temperature and isothermal annealing time on the structure, tribological properties and corrosion behavior of ADI cast iron.

## 2. Materials and Methods

### 2.1. Material Prepartion

For the tests, ductile cast iron (DI) was used. The chemical composition of DI is presented in Table 1. To obtain austempered ductile iron (ADI), cast iron cylinder-shaped ductile iron samples with a diameter of 10 mm and length of 100 mm were quenched with isothermal transformation. The samples were austenitized in a resistance furnace at 900 °C for a period of 2 h and then quenched to the isothermal temperature in a salt bath. Two extreme values of austempering temperatures were selected, i.e., 280 °C and 430 °C, representing the lower and upper ausferrite, respectively. The austempering time at both of these temperatures was the same and amounted to 30 and 120 min. For further tests, it was decided to determine the samples 2.1 and 6.1 for the austempering temperature of 280 °C and the isothermal annealing times of 30 and 120 min, respectively, and the 2.2 and 6.2 samples for the austempering temperature of 430 °C and the isothermal annealing times of 30 and 120 min, respectively.

### 2.2. Microstructure Research

Microstructure tests were carried out on metallographic specimens cut from samples of ADI cast iron produced in four variants. All the specimens were mechanically ground with emery papers down to 4000 grit and polished with diamond pastes down to 1 μm. Between each step of grinding and polishing, the specimens were ultrasonically rinsed in ethanol for 5 min. Metallographic specimens were etched with 4% nital (i.e., a 4% solution of nitric acid in methanol). In addition, in order to identify the phase components of the microstructure, the samples were subjected to color etching using the B-M reagent (i.e., 100 mL of the basic solution, 2 g of NH_4_F HF and 2 g of K_2_S_2_O_5_, where the basic solution consisted of 100 mL of distilled water and 20 mL of HCl) [44].

Observations of the microstructure of the ADI cast iron were made using the Neophot 2 optical microscope Carl Zeiss, Jena, Germany, equipped with the MultiScan image acquisition and processing system.

Observations of the ADI cast iron microstructure were made using a Vega scanning electron microscope, made by Tescan, equipped with an X-act microanalysis adapter (SEM, TESCAN, Brno, Czech Republic).

Microhardness measurements were performed on a ZHVμ Indentec microhardness tester, manufactured by Zwick/Roell, using a load of 25 g (F = 0.245 N), Ulm, Germany. Nine measurements were made on each sample and the arithmetic mean was taken.

### 2.3. Scratch Tests

Scratch resistance tests of the ADI cast iron samples were performed using the Scratch Tester Revetest RST device, manufactured by CSM Instruments (Peseux Switzerland). The scratches were made with a Rockwell-type diamond indenter with a tip radius of 200 µm and an angle of 120°. A loading force of 10 N was used. The length of the scratch was 2 mm. The observations of cracks were made using a Vega scanning microscope, from Tescan.

In the scratch test, the value of the friction force, Ff, the friction coefficient, FC, the penetration depth, Pd, and the value of the acoustic emission signal index, AE, were determined. The value of the acoustic emission signal, AE, recorded during the creation of the scratch was given as a percentage in relation to the acoustic emission signal of the standard, i.e., titanium nitride (TiN), for which the signal value AE = 65 dB was taken as 100%.

### 2.4. Corrosion Tests

The corrosion resistance of the ADI specimens was determined in a 0.05 M NaCl solution. Linear sweep voltammetry (LSV) tests were performed for all specimens in sodium chloride solutions. The corrosion test was performed in a classical three-electrode electrochemical cell containing silver–silver chloride Ag/AgCl (3 M KCl) as a reference electrode, a platinum plate as a counter electrode and the specimen used as the working electrode. The LSV curves were plotted from −800 mV vs. Ag/AgCl (3 M KCl) to the anodic direction with the potential scan rate of 1 mV/s. The evolution of open circuit potential (OCP) in the sodium chloride solution was monitored for 24 h for all specimens.

Electrochemical impedance spectroscopy was used to study the corrosion behavior of the ductile iron specimens. EIS spectra were measured in the frequency range from 10 kHz up to 8 mHz. The amplitude of the potential perturbation signal was 10 mV. The spectra were measured at the open circuit potential. The EIS data was fitted by using ZView 4 software. The electrochemical experiments were performed with a Metrohm Autolab PGSTAT128 Potentiostat/Galvanostat (Herisau, Switzerland) and the Nova 2.1 software.

## 3. Results and Analysis

### 3.1. Microstructure Research Results

**Figure 1:**
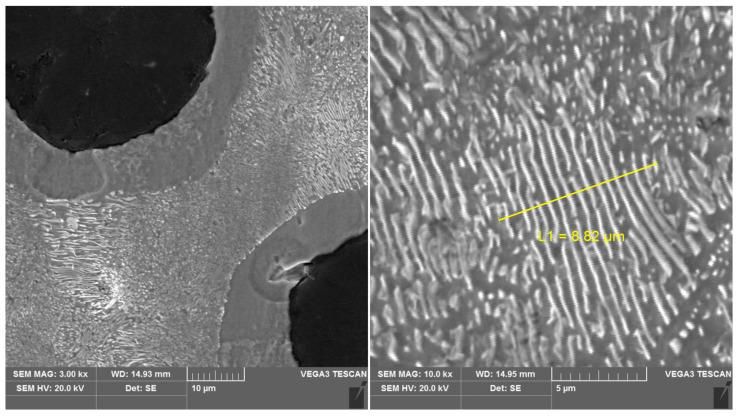
Microstructure of ductile iron before heat treatment of austempering.

The starting material for further research was ductile cast iron, the microstructure of which is shown in Figure 1. As can be seen, the structure of the starting cast iron was characterized by a pearlitic–ferritic structure with a pearlite dispersion of 0.55 μm.

**Figure 2:**
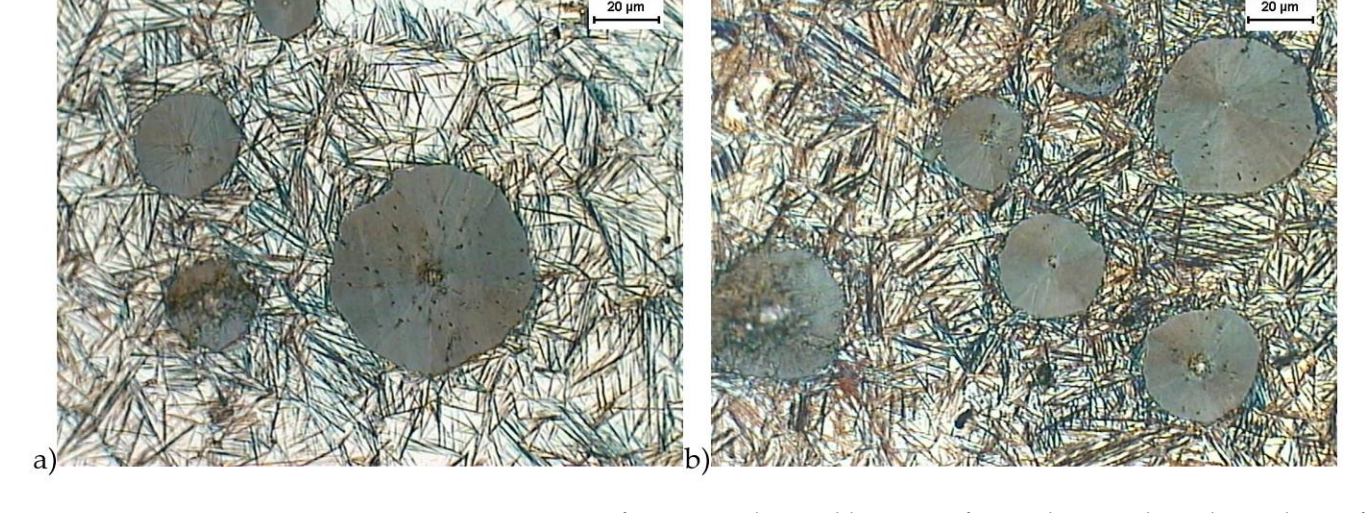
Microstructure of cast iron obtained because of quenching with isothermal transformation of ductile iron to the austempering temperature, T_austempering_ = 280 °C, austempering time, *τ*_austempering_ = 30 min (**a**) and 120 min (**b**), etched with 4% nital reagent.

**Figure 3:**
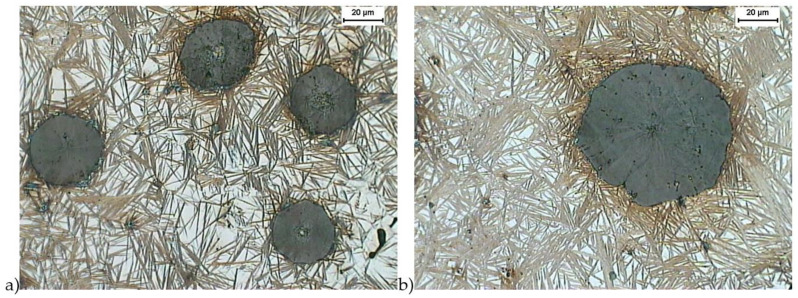
Microstructure of cast iron obtained because of quenching with isothermal transformation of ductile iron to the austempering temperature, T_austempering_ = 430 °C, austempering time, *τ*_austempering_ = 30 min (**a**) and 120 min (**b**), etched with 4% nital reagent.

Figure 2 and Figure 3 show the structure of the ductile iron after quenching, including isothermal transformation of the ductile iron. The obtained results show the morphology of ausferrite plates, a mixture of high-carbon austenite and ferrite. The lower ausferrite plates (with an austempered temperature of 280 °C, Figure 2) were deposited thinner than the upper ausferrite plates (with an austempered temperature of 430 °C, Figure 3). This difference in ausferrite thickness will affect the mechanical properties of this material. In addition, the X-ray analysis showed that the high-carbon austenite contained more than 2% carbon. Such a high carbon content in the austenite reduced the martensite start to –120 °C [6,7], causing the stabilization of the austenite in the structure of the ADI cast iron at room temperature.

**Figure 4:**
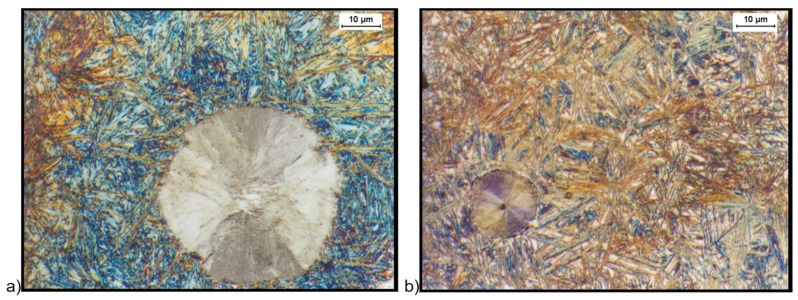
Microstructure of cast iron obtained because of quenching with isothermal transformation of ductile iron to the austempering temperature, T_austempering_ = 280 °C, and austempering time *τ*_austempering_ = 30 min, (**a**) and 120 min (**b**), etched with B-M reagent.

**Figure 5:**
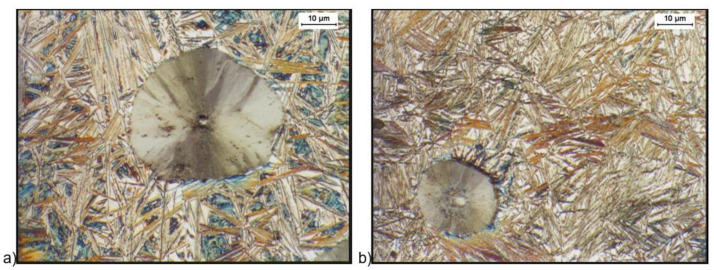
Microstructure of cast iron obtained because of quenching with isothermal transformation of ductile iron to the austempering temperature, T_austempering_ = 430 °C, and austempering time, *τ*_austempering_ = 30 min (**a**), and 120 min (**b**), etched with B-M reagent.

Figure 4 and Figure 5 show the structure of the ductile iron after quenching including the isothermal transformation of ductile iron etched with the B-M reagent. The B-M reagent colors martensite blue and ausferrite brown but does not color high-carbon austenite and carbides [44]. The obtained results show that the most martensite (blue color) was found in the sample hardened with isothermal transformation to the temperature of 280 °C and isothermally annealed for 30 min (Figure 4a). Much less martensite was observed in a sample isothermally annealed for 30 min at a temperature of 430 °C (Figure 5a). For samples hardened with isothermal transformation for both the temperature values, i.e., 280 °C (Figure 4b) and 430 °C (Figure 5b) for 2 h, the presence of martensite was not observed.

**Figure 6:**
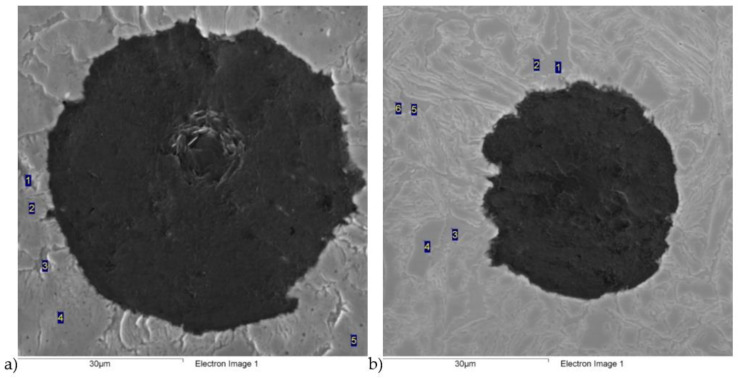
Scanning electron microscopic images of ductile iron austempered at a 280 °C temperature for different austempered time: (**a**) 30 min; (**b**) 120 min.

**Figure 7:**
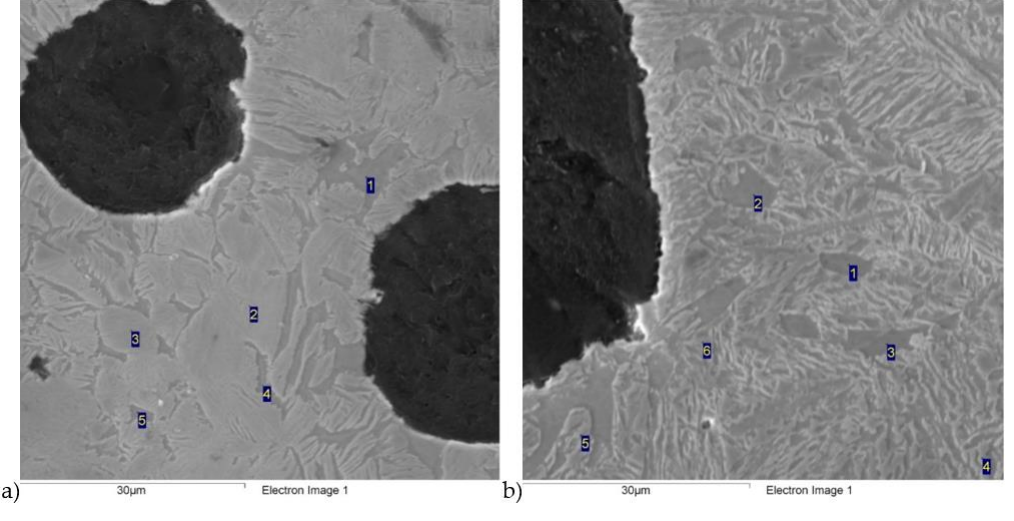
Scanning electron microscopic images of ductile iron austempered at a 430 °C temperature for different austempered time: (**a**) 30 min; (**b**) 120 min.

Scanning electron microscope (SEM) tests were carried out, consisting of a point analysis of the chemical composition of several areas, shown in Figure 6 and Figure 7. The results of this analysis are presented in Table 2, Table 3, Table 4 and Table 5. The results of the analysis show that higher concentrations of silicon were observed in the ferrite than in the austenite, and carbon segregation behaved in a completely different way, with the highest concentration observed in the austenite. In addition, it is evident that with the extension of the austempering time, the carbon concentration in the austenite also increased.

### 3.2. Scratch Test Results

**Figure 8:**
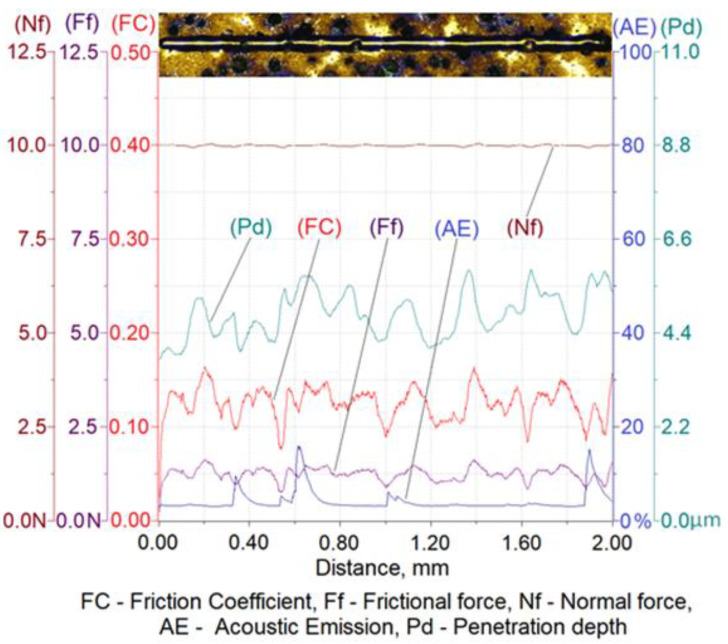
Results of ADI cast iron scratch susceptibility tests in variant 2.1.

**Figure 9:**
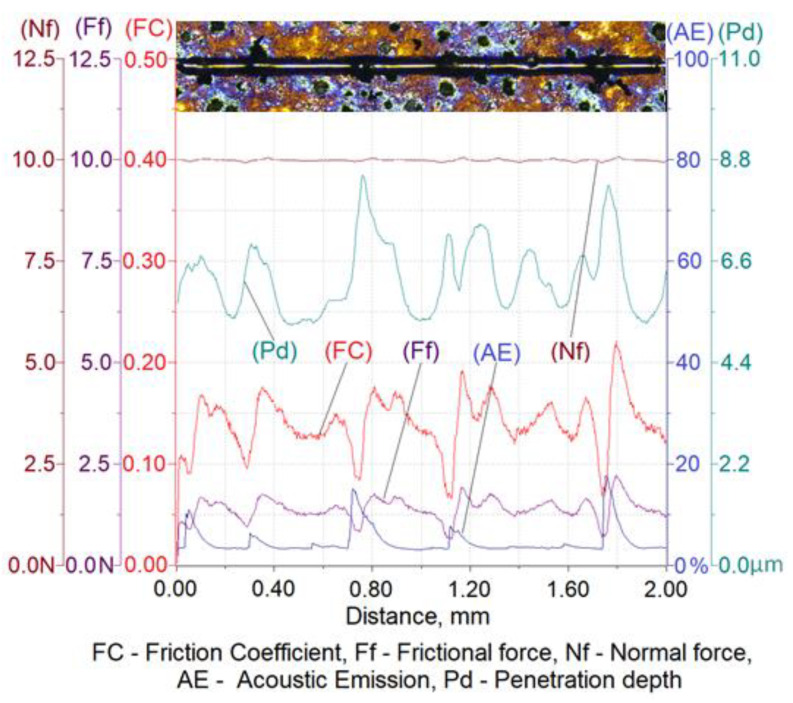
Results of ADI cast iron scratch susceptibility tests in variant 6.1.

**Figure 10:**
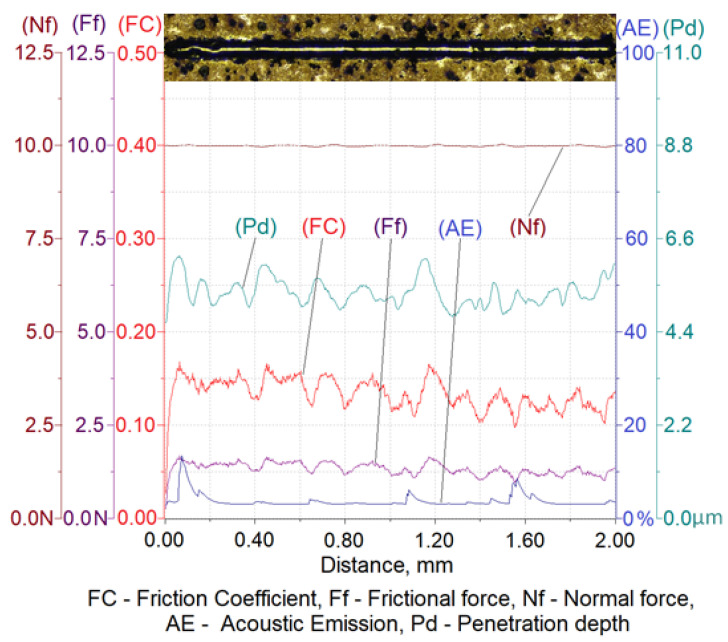
Results of ADI cast iron scratch susceptibility tests in variant 2.2.

**Figure 11:**
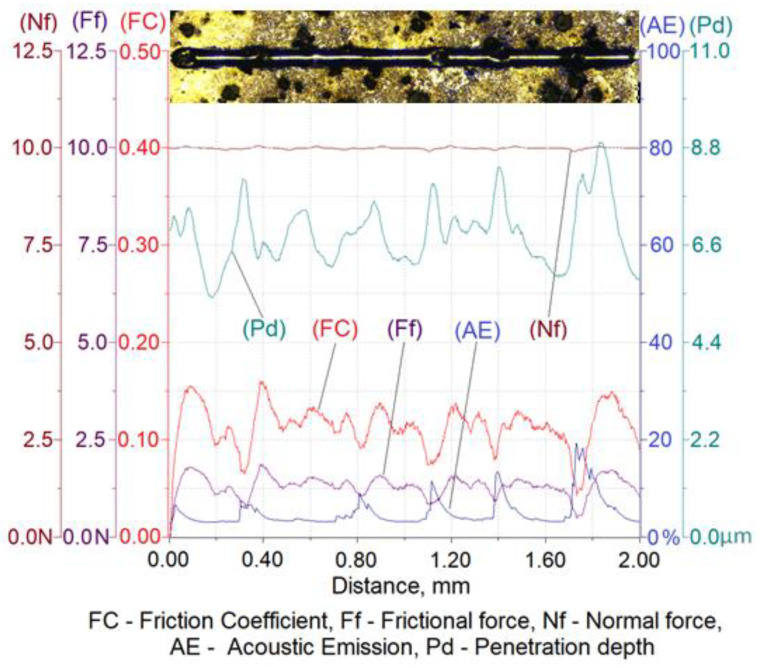
Results of ADI cast iron scratch susceptibility tests in variant 6.2.

The results of a susceptibility to crack formation under the action of a diamond indenter on samples from individual variants of ADI cast iron are shown in Figure 8, Figure 9, Figure 10 and Figure 11.

Table 6 shows the ranges of variability and the average values of the friction force of Ff, the depth of cracks of Pd, the friction coefficient of FC and the values of the acoustic emission index of AE made on four variants of ADI cast iron samples.

The analysis of the scratch susceptibility test results shows that the extension of the austempering time of the cast iron from 30 min (variant 2.1) to 120 min (variant 6.1) at the temperature of 280 °C increased the scratch depth while the hardness of the samples decreased from 492 HV0.025 to 475 HV0.025, respectively. A similar set of changes in the scratch depth was observed in the case of the austempering of cast iron at 430 °C. With an increase in the austempering time from 30 min (variant 2.2) up to 120 min (variant 6.2), the scratch depth increased and the hardness of the samples decreased from 427 HV0.025 to 414 HV0.025, respectively. The average value of the scratch depth of the samples annealed isothermally at 430 °C was slightly higher than the depth of cracks annealed isothermally at 280 °C, but the hardness was slightly lower for the samples annealed at 430 °C than for 280 °C. The increase in the scratch depth along with the extension of the annealing time was associated with a change in the structure. During isothermal annealing, the process of nucleation and the growth of ferrite plates takes place, because the maximum solubility of carbon in ferrite is very small in relation to austenite; therefore the crystallization of ferrite pushes the carbon to austenite. An increase in the carbon content to about 2% in austenite reduces the martensite start temperature down to −120 °C [6,7]. Thanks to this phenomenon, austenite becomes stable at ambient temperatures, while austenite containing a smaller amount of carbon transforms into martensite, which has a higher hardness. This is due to the fact that the higher the value of the austempering temperature, the faster the diffusion of carbon leading to the austenite becoming saturated with carbon more quickly, thus causing its stabilization at room temperature. This means that the less martensite in the cast iron, the deeper the scratch depth. This phenomenon was confirmed by the hardness measurement results and the microstructure obtained as a result of the color etching, as shown in Figure 4 and Figure 5.

With an austempering temperature at 280 °C, the value of the friction coefficient increased with the increase in the isothermal annealing time, while in the case of the austempering temperature of 430 °C, the value of the friction coefficient decreased with the increase in the isothermal annealing time. This phenomenon was associated, on the one hand with the presence of martensite and, on the other hand, with the carbon content in the martensite, because the more carbon that the martensite contains, the more distorted the lattice and the greater the hardness. In the case of the cast iron heated at 280 °C, the process of carbon diffusion to austenite occurred, causing its carburization. Due to the low austempering temperature, the carbon diffusion was slow and the carbon content in the austenite, even after 120 min, was not at a sufficient level that would allow a stabilization of the austenite at room temperature. Therefore, the structure will have contained martensite with an increasing concentration in carbon, resulting in an increase in the hardness and, thus, an increase in the coefficient of friction. The situation may have been slightly different in the case of annealing the cast iron at the temperature of 430 °C, where the carbon diffusion will have proceeded much faster and, consequently, the austenite may have turned into residual austenite after 120 min, which had a lower hardness than the martensite. By contrast, for the same isothermal annealing temperature but for an austempering time of 30 min, the structure would contain martensite, which is harder than the retained austenite and, thus, the structure would have a high coefficient of friction.

**Figure 12:**
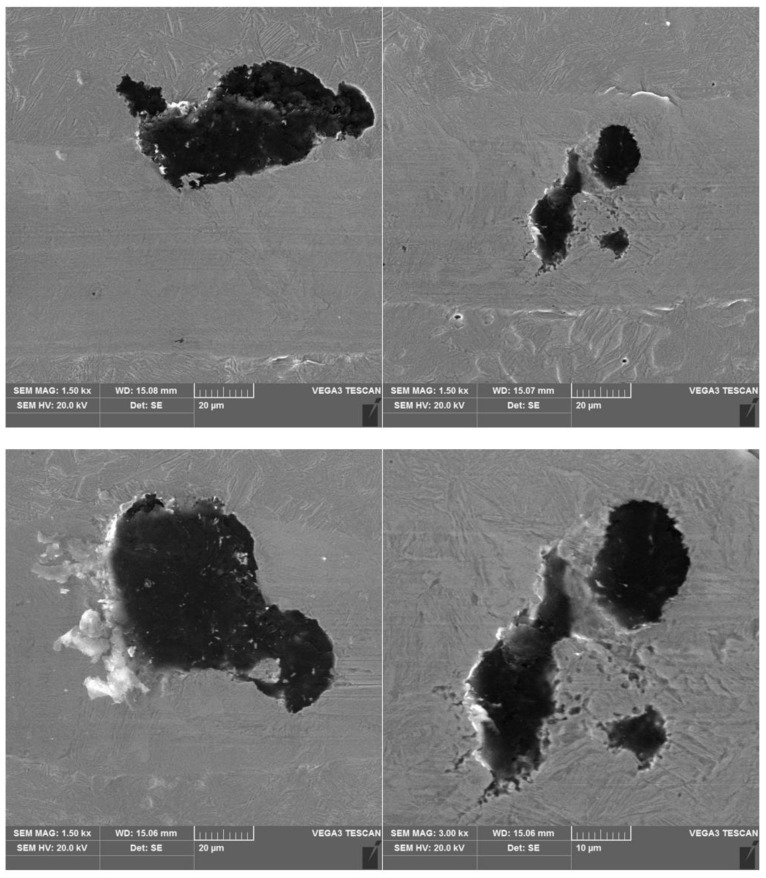
SEM images of a scratch made on an ADI cast iron sample—variant 2.1.

**Figure 13:**
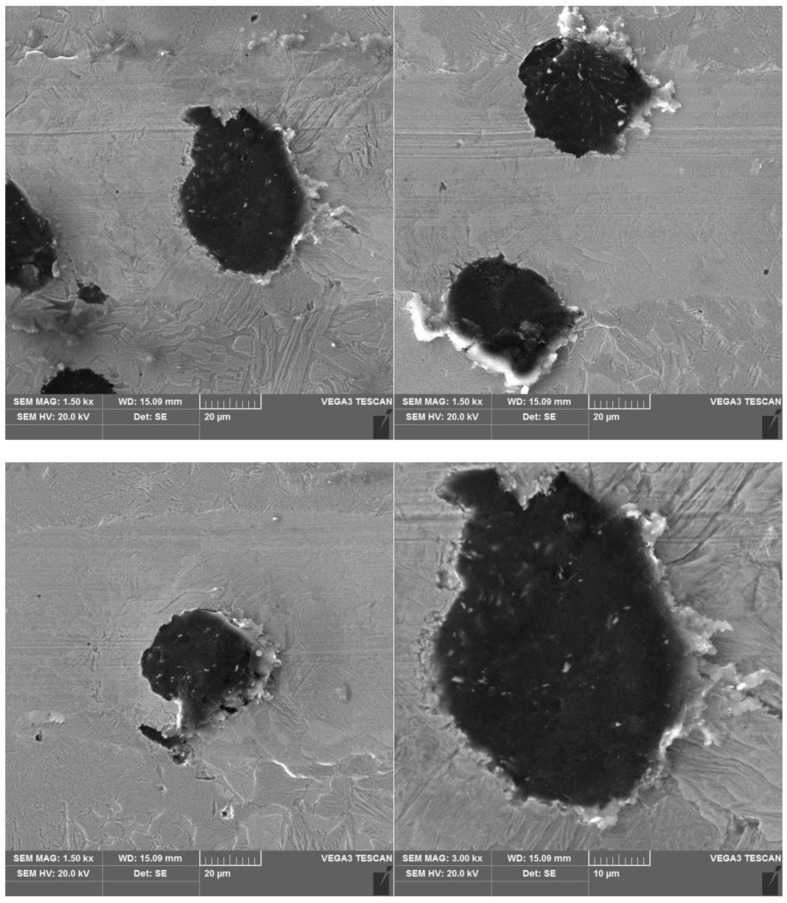
SEM images of a scratch made on an ADI cast iron sample—variant 6.1.

**Figure 14:**
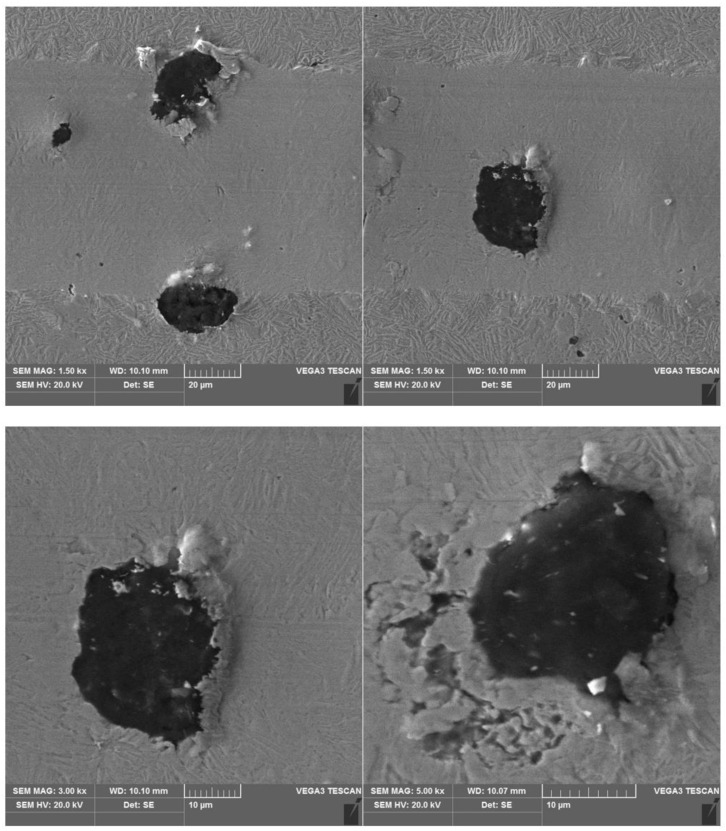
SEM images of a scratch made on an ADI cast iron sample—variant 2.2.

**Figure 15:**
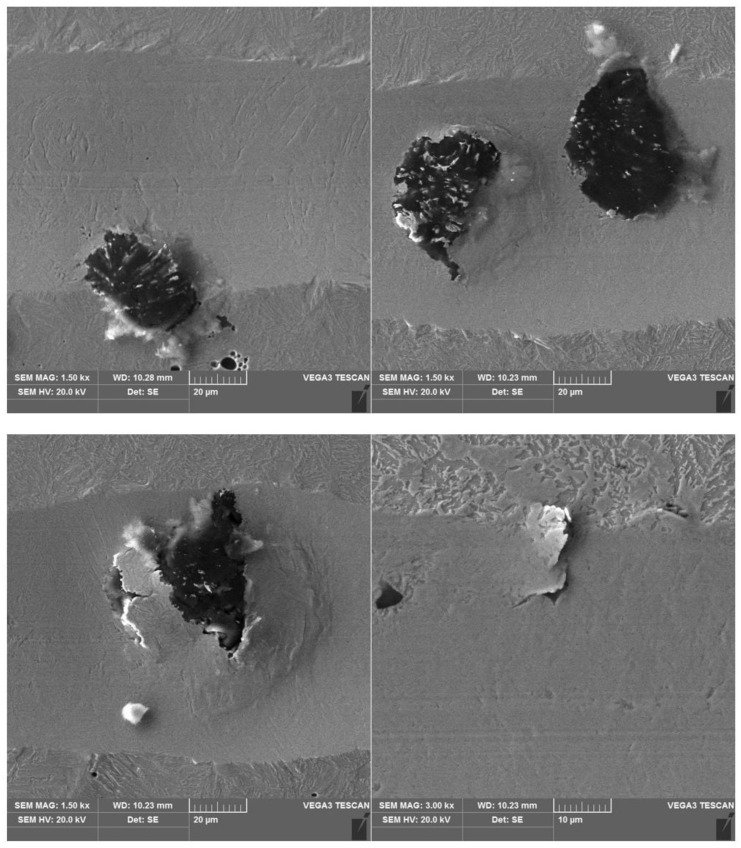
SEM images of a scratch made on an ADI cast iron sample—variant 6.2.

The peaks of the acoustic emission signal observed in the graphs (Figure 8, Figure 9, Figure 10 and Figure 11) testify to the response of the material during its drawing to the action of the diamond indenter. However, the analysis of the SEM images of the scratch area of the individual ADI cast iron variants did not show the presence of cracks (Figure 12, Figure 13, Figure 14 and Figure 15); therefore, it can be assumed that the peaks of the AE signal may have originated from delaminations of the matrix at the graphite-matrix interface, caused by the passage of the diamond indenter through the graphite precipitates.

### 3.3. Corrosion Resistance of Ductile Iron

#### 3.3.1. Corrosion Rate of Ductile Iron

**Figure 16:**
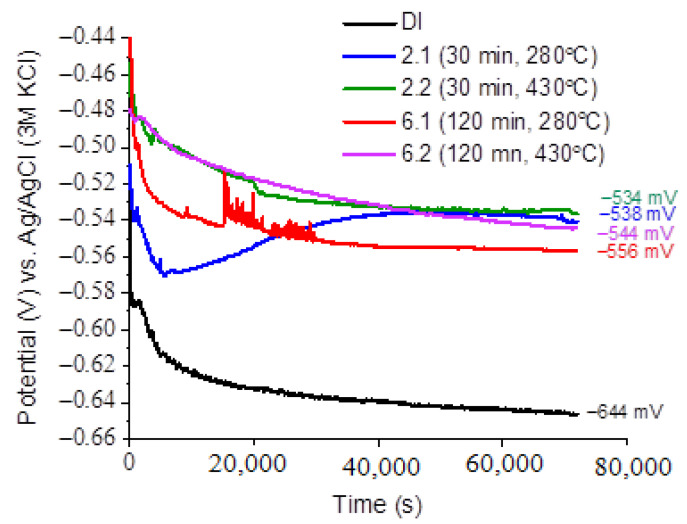
Evolution of open circuit potential (OCP) versus time in 0.05 M NaCl solution.

**Figure 17:**
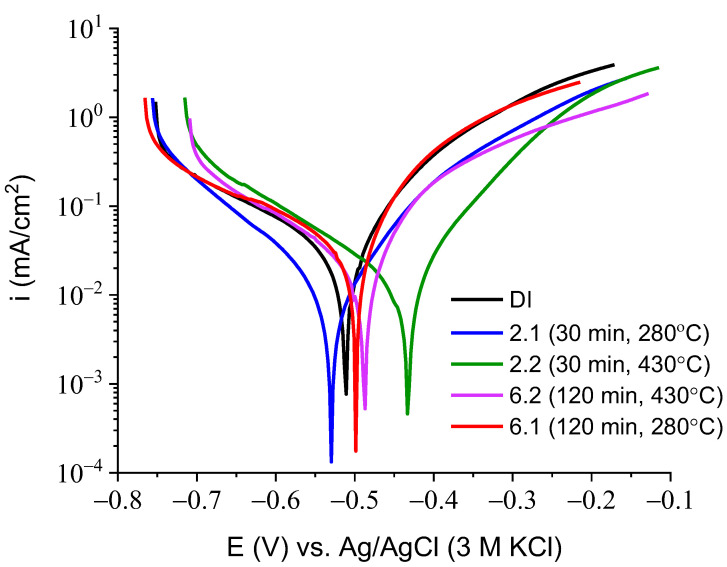
LSV curves measured for ductile iron in the 0.05 M NaCl solution. Potential scan rate of 1 mV/s.

Figure 16 shows the evolution in the open circuit potential over time, measured for all samples in a sodium chloride solution. The potential values for all alloys decreased after their immersion in the corrosive solution. After an immersion longer than 20 h, the potential reached a stable value for all alloys. The lowest potential value was recorded for the DI sample, after 24 h of exposure in the sodium chloride solution, at −646 mV (black curve, Figure 16). The highest potential value of −533 mV was measured for the sample labelled 2.2 (green curve, Figure 16). This result suggests that the 2.2 specimen should show less susceptibility to corrosion compared to the other specimens. Note that for sample 6.1 (red curve, Figure 16), potential oscillations were evident in the interval time from 5 to 10 h of immersion of the sample in the solution. Such oscillations were associated with a perturbation of the passive film or the initiation of pitting corrosion. The course of potential changes over time indicates that all the alloys tended to undergo active corrosion in a 0.05 M sodium chloride solution. Figure 17 shows the polarization curves made for all alloys in a sodium chloride solution. In the cathodic branch, the highest current density values were recorded for sample 2.2 (green curve, Figure 17). This indicates that the cathodic oxygen reduction reaction (ORR), namely, Reaction (1), was favored on the surface of sample 2.2:

(1) $$O_{2}+2H_{2}O+4e=4{\mathrm{OH}}^{-}$$

The presence of OH^−^ ions led to alkalization of the sample surface, and the corrosion potential was shifted towards the anodic direction, (green curve, Figure 17). The lowest values of cathodic current density were recorded for sample 2.1 (blue curve, Figure 17). In contrast, there were no significant differences in the current density recorded in the cathodic region for the other samples (i.e., DI, 6.1, and 6.2).

For the samples tested, significant differences in the current density values were observed in the anodic region. The highest values of anodic current density were recorded for the samples DI and 6.1 (black and red curves, respectively, Figure 17). In contrast, the lowest values of anodic current were registered for sample 2.2, indicating a slower corrosion of this sample compared to the other samples. The anodic current density was related with the dissolution of iron associated with Reaction (2):

(2) $$\mathrm{Fe}={\;\mathrm{Fe}}^{2+}+2e$$

From the polarization curves, as shown in Figure 17, such parameters as the corrosion potential and corrosion current were determined. The corrosion rate for each sample was calculated using Equation (3):

(3) $$CR=\frac{i_{corr}\cdot K\cdot EW}{d\cdot A}$$

where *CR* is the corrosion rate expressed in millimeters per year (mm/y), *i_corr_* is the corrosion current expressed in amperes, *EW* is the equivalent weight of the corroding metal in grams, *d* is the density of the corroding metal in g/cm^3^, and *A* is the surface of the specimen expressed in cm^2^.

The values of corrosion potential, corrosion current and corrosion rate are given in Table 7. As shown in Table 7, the corrosion rate of the ductile iron (DI) alloy was more than twice that of the alloys marked as 2.1 and 2.2. The lowest value of corrosion rate of the specimens 2.1 and 2.2 were attributed to the presence of martensite in the microstructure.

#### 3.3.2. EIS Measurements

**Figure 18:**
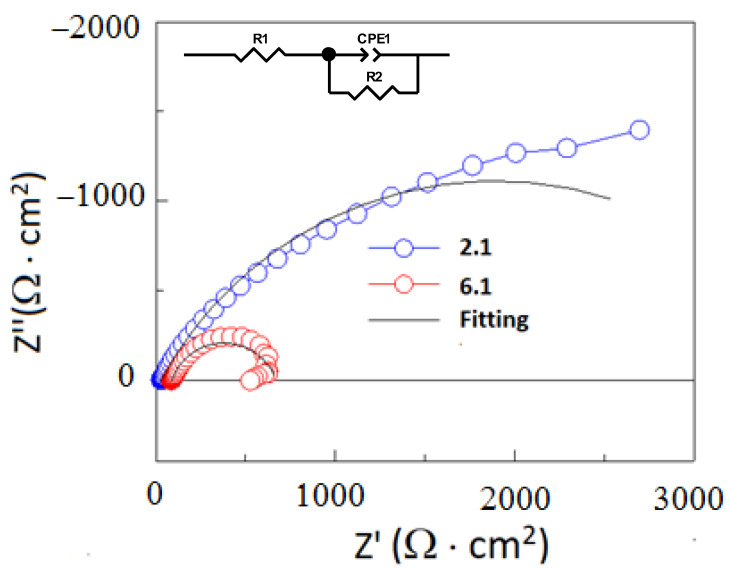
EIS spectra performed for 2.1 and 6.1 specimens at OCP in a 0.05 M NaCl solution. The electrical equivalent circuit.

The differences in the corrosion resistance of the ductile iron specimens were verified by EIS measurements. The EIS spectra were performed for specimens 2.1 and 6.1. These specimens were austempered at 280 °C with the isothermal annealing time at 30 and 120 min for 2.1 and 6.1, respectively. The EIS spectra and the electrical equivalent circuit used for the fitting of the experimental data are presented in Figure 16. The data of the fitting parameters are given in Table 8. The EIS diagrams were fitted using a resistance R1 (electrolyte resistance) connected in series with a constant phase element CPE1 and a parallel R2 resistance of the oxide layer formed at the surface of the austempered ductile iron. The constant phase element (*CPE*) replaced an ideal capacitor for a non-ideal capacitance of the electrode, and its impedance (*Z_CPE_*) is given by Equation (4) [45]:

(4) $$Z_{CPE}={\left[{\left(j\omega \right)}^{p}T_{0}\right]}^{-1}$$

where *j* is the imaginary number, *ω* is the angular frequency input in the EIS test, *T*_0_ is the admittance, and *p* is the phenomenological coefficient, indicating the deviated degree of the ideal capacitor. The value of *p* can be in the range between 0 and 1. If *p* is equal to 1, *CPE* represents an ideal capacitor. When the *p* is equal to 0, the *CPE* represents a resistor. When *p* equals 0.5, the *CPE* behaves as a Warburg impedance and the diffusion process takes place at the interface of the metal/electrolyte. The EIS spectra obtained for the 2.1 and 6.1 specimens exhibited the presence of one capacitive loop, as shown in Figure 18. The highest values of the resistance R2 was measured for the 2.1 specimens, which indicated a higher corrosion resistance than for the 6.1 specimens. The value of the *CPE-P* for these specimens was around 0.7 and 0.8 for the 2.1 and 6.1 specimens, respectively. These values indicate that the capacitive character of the passive film formed at the surface of specimens 2.1 and 6.1.

#### 3.3.3. Corrosion Products under Free Corrosion (OCP)

**Figure 19:**
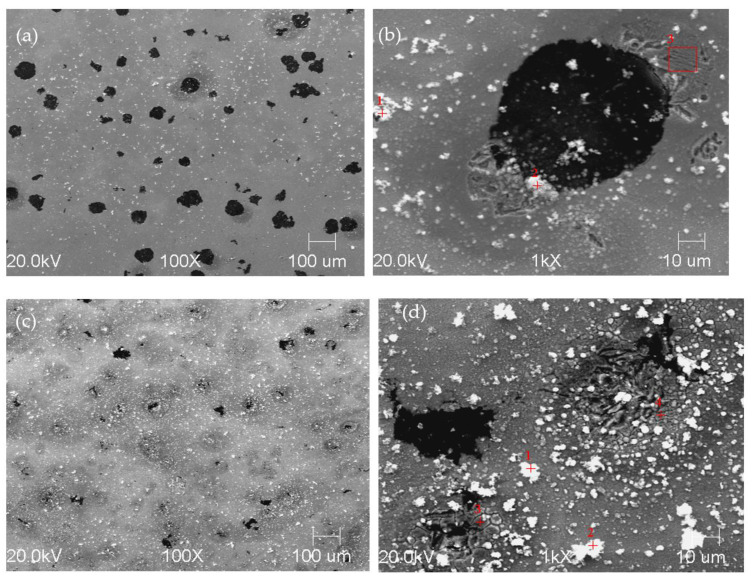
SEM images of specimen 2.1 (**a**,**b**) and 6.1 (**c**,**d**) revealed after exposition of specimens in 0.05 M NaCl solution for 1 h.

**Figure 20:**
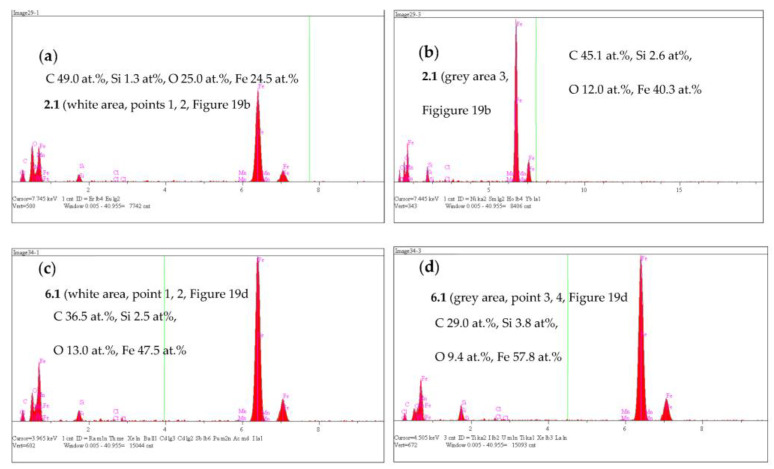
EDS spectra of corrosion products formed at 2.1 specimen (**a**,**b**) and 6.1 (**c**,**d**).

To prove that the presence of martensite had a relevant effect on the corrosion rate, samples 2.1 and 6.1 were immersed in a 0.05 M NaCl solution for 1 h, after which surface observations were performed on both alloys. Figure 19 shows photographs of the surfaces of the alloys 2.1 and 6.1 after 1 h in the corrosive environment. It is clearly noticeable that sample 6.1 was more corroded than sample 2.1, as shown in Figure 19. Note that the corrosion of both samples started at the graphite/matrix interface. The corrosion products mainly contained carbon, oxygen, silicon, and iron. The chemical composition of the corrosion products for both samples is given in Figure 20.

## 4. Discussion

In conclusion, it can be determined that due to the high content of silicon in ductile cast iron, iron carbide does not precipitate immediately, as is the case with steel. At the isothermal transformation temperature of 280 °C, the growth rate of ferrite plates is high, and because the carbon diffusion rate is relatively low, it leads to the supersaturation of bainitic ferrite [1]. Due to the excess of carbon, this phase may initially have a distorted tetragonal crystal lattice. From the bainitic ferrite, excess carbon is removed to the adjacent austenite, with the transformation taking place continuously. This process leads to an increase in the carbon concentration in the austenite until the value reaches about 2%. Then, after the end of the isothermal transformation and after cooling down to ambient temperature, the share of retained austenite is small and may amount to about 10% [5]. Such a structure, referred to as lower ausferrite [1], is characterized by fine precipitates of ferrite with a small amount of retained austenite.

At the isothermal transformation temperature of 430 °C, a slightly different transformation mechanism takes place. Carbon diffusion is faster, so that more carbon can be diffused from the growing ferrite plates, enriching the remaining austenite, especially between the growing ferrite plates. If the isothermal process is stopped early, the martensitic onset temperature (Ms) will remain above ambient temperature, and the remaining austenite will convert at least partially to martensite on cooling. If the isothermal holding time is further extended, the carbon content of the remaining austenite will increase and eventually reach a level of approximately 1.5 to 1.7% [5]. Despite such a high level of carbon content, which may cause the thermal stabilization of austenite, under the influence of loads (e.g., during mechanical processing) it may transform into martensite [6,7]. In the literature this phenomenon is called SATRAM [46]. Further extension of the isothermal transformation time to 2 h results in the continued growth of ferrite plates, as a result of which the austenite is further enriched in carbon, reaching the level of 1.8–2.2% [6,7]. Such a high carbon content in austenite lowers the onset of the martensitic temperature (Ms). The result of this phenomenon is the presence of residual austenite in the structure after cooling to ambient temperature. This retained austenite is stable down to −120 °C, and the structure of ductile iron after isothermal transformation will consist mainly of thick plates of ferrite and retained austenite. This second phase can reach up to 40% of the matrix [5,10], and this structure is called upper ausferrite [1]. The presence of such a large amount of retained austenite is responsible for the high level of ductility and hardness of ADI cast iron.

The morphology of the ferrite plates and the segregation of such elements as carbon and silicon affect both the mechanical [5,47], tribological and corrosion properties.

Therefore, an isothermal annealing time of 30 min (observed in sample 2.1) for an isothermal austempering temperature of 280 °C is too short to allow a 100% conversion of austenite to ausferrite. Similar results were observed in earlier studies [39], where for cast iron of the same chemical composition and annealed at the same temperature for only 10 min, a structure consisting of graphite spheres, martensite, and a small amount of ausferrite was obtained. In this case, the isothermal annealing time was only slightly longer. This may only result in a slightly higher share of ausferrite in relation to martensite. A similar structure (martensite–ausferrite) will be characteristic of a sample annealed at 430 °C for 30 min. A slight difference in the structure (i.e., the martensite to ausferrite proportion) will result from a higher austempering temperature. A higher value of the isothermal annealing temperature will result in a higher diffusion rate of carbon to austenite and, thus, a greater share of ausferrite in the sample structure in relation to martensite, compared to a sample annealed for 30 min at 280 °C; however, for both values of the austempering temperature, isothermally annealed for 120 min, the structure of the samples will consist of ausferrite. The only difference will be in the thickness of the ausferrite plates. In the case of a higher austempering temperature, the ausferrite plates will be thicker than for a lower austempering temperature. This difference both in the composition of the phase components of the structure, and in the morphology, affects both the tribological and corrosion properties. Therefore, in the case of samples annealed isothermally at both 280 and 430 °C for 30 min, the scratch depth will be smaller than for longer annealing times (120 min). This is related to the presence of martensite in the matrix of the samples. This presence of martensite in the cast iron structure also makes it more resistant to corrosion. This phenomenon is related to the fact that the specific volume of martensite is about 2% greater than the specific volume of austenite. This causes the generation of compressive stresses in the austenite. It is well known that materials in which compressive stresses occur in the structure are characterized by better corrosion resistance [48,49,50]. In general, it has been shown that under compression, the chemical composition of the passive layer and its thickness change. The presence of compressive stresses eliminates or reduces the occurrence of pores, crevices and, as a result, the passive layer is more compact, providing better protection of the material against corrosion. In addition, it should be noted that austenite has a face-centered, regular A1 crystal lattice, ferrite has a spatially-centered, cubic A2 crystal lattice, and martensite has a spatially-centered tetragonal lattice. Therefore, the spatially-centered regular lattice is the most loosely packed crystal structure. This results in the easiest removal of atoms from the crystal lattice and this may result in faster corrosion in the ferrite.

## 5. Conclusions

The scratch depth of the cast iron samples subjected to an austempering heat treatment increases with the extension of the isothermal annealing time (from 30 to 120 min) and austempering temperature (from 280 °C to 430 °C).

Extending the isothermal annealing time from 30 to 120 min causes an increase in the fraction of ausferrite structures in relation to martensite.

Increasing the austempering temperature from 280 °C to 430 °C increases the transformation kinetics and, at the same time, affects the morphology of the final ausferrite structure.

The presence of acoustic signal emission peaks come from matrix delaminations at the graphite-matrix interface.

The change in the structure of cast iron during the austempering of ductile iron affects the corrosion resistance. Martensitic cast iron has the best corrosion resistance. This is due to an increase in the specific volume of martensite in relation to the specific volume of austenite. In addition to this, ferrite, due to a crystal lattice structure, is characterized by a loose packing of atoms in the crystal lattice in relation to austenite. This structure of the crystal lattice causes faster pitting corrosion of the ferrite.

## Figures and Tables

**Table 1:** Chemical composition of ductile iron.

C, wt.%	Mn, wt.%	Si, wt.%	P, wt.%	S, wt.%	Cr, wt.%	Ni, wt.%	Cu, wt.%	Mg, wt.%	Mo, wt.%	Ti, wt.%	Sn, wt.%	Pb, wt.%	V, wt.%	W, wt.%	Zn, wt.%
3.48	0.63	2.56	0.03	0.017	0.06	0.04	0.32	0.054	0.07	0.015	0.006	0.001	0.009	0.038	0.003

**Table 2:** Point analysis of chemical composition for Figure 4a.

Number	C, wt.%	Si, wt.%	Mn, wt.%	Fe, wt.%	Cu, wt.%
1	1.25	3.38	0.20	94.78	0.38
2	2.11	2.79	0.47	94.35	0.28
3	1.20	3.99	0.21	94.32	0.27
4	2.09	3.13	0.52	93.69	0.57
5	2.13	2.95	0.35	94.03	0.54

**Table 3:** Point analysis of chemical composition for Figure 4b.

Number	C, wt.%	Si, wt.%	Mn, wt.%	Fe, wt.%	Cu, wt.%
1	1.11	4.03	0.08	94.31	0.47
2	1.94	2.96	0.30	94.34	0.46
3	0.90	4.07	0.02	94.74	0.27
4	2.75	2.95	0.32	93.45	0.52
5	0.98	3.70	0.11	94.65	0.56
6	2.30	2.80	0.44	94.09	0.37

**Table 4:** Point analysis of chemical composition for Figure 5a.

Number	C, wt.%	Si, wt.%	Mn, wt.%	Fe, wt.%	Cu, wt.%
1	1.39	3.79	0.21	94.23	0.39
2	2.91	3.12	0.23	93.30	0.44
3	2.94	2.50	0.51	93.80	0.25
4	1.21	3.58	0.23	94.58	0.40
5	1.00	3.86	0.14	94.53	0.47

**Table 5:** Point analysis of chemical composition for Figure 5b.

Number	C, wt.%	Si, wt.%	Mn, wt.%	Fe, wt.%	Cu, wt.%
1	1.18	3.97	0.15	94.49	0.21
2	1.31	4.25	0.04	93.99	0.41
3	1.11	3.94	0.16	94.62	0.18
4	3.77	2.60	0.29	93.08	0.25
5	4.71	2.51	0.28	92.08	0.42
6	3.92	2.99	0.37	92.26	0.46

**Table 6:** Values of scratch depth, Pd, friction force, Ff, friction coefficient, FC and acoustic emission signal index, AE of scratches made on four variants of ADI cast iron.

Parameter		Sample Variant			
		2.1	6.1	2.2	6.2
Pd, μm	minimum	3.91	5.23	4.77	5.41
	maximum	5.89	8.47	6.20	8.93
	average	4.90	6.85	5.49	7.17
Ff, N	minimum	0.77	0.67	0.98	0.52
	maximum	1.64	2.22	1.64	1.86
	average	1.20	1.44	1.31	1.19
FC	minimum	0.077	0.067	0.098	0.052
	maximum	0.164	0.222	0.164	0.186
	average	0.120	0.144	0.131	0.119

**Table 7:** Data calculated from the electrochemical measurements (LSV curves).

Specimen	Ecorr [mV]	Icorr [A]	CR [mm/y]
DI	−513	2.4·10^−5^	0.36
2.1	−523	1.0·10^−5^	0.15
2.2	−451	1.0·10^−5^	0.15
6.1	−502	2.1·10^−5^	0.31
6.2	−492	1.7·10^−5^	0.25

**Table 8:** Data of the fitting parameters obtained for the EIS diagrams of 2.1 and 6.1 specimens.

	Sample 2.1	Sample 6.1
R1 [Ω·cm^2^]	29.0 ± 0.35	38.0 ± 0.4
CPE1-T [Ω^−1^·cm^−2^·s^p^]	0.00102 ± 1.7×10^−5^	0.00091 ± 1.6×10^−5^
CPE1-P	0.7 ± 0.005	0.77 ± 0.006
R2 [Ω·cm^2^]	3087 ± 114	621 ± 7.8

## Data Availability

The data presented in this study are available on request from the corresponding author.
